# Editorial: The Immunobiology of HLA-Haploidentical Hematopoietic Cell Transplantation

**DOI:** 10.3389/fimmu.2020.01031

**Published:** 2020-05-20

**Authors:** Antonella Mancusi, Christopher G. Kanakry, Antonio Pierini

**Affiliations:** ^1^Hematology and Clinical Immunology and Bone Marrow Transplant Program, Department of Medicine, University of Perugia, Perugia, Italy; ^2^Experimental Transplantation and Immunotherapy Branch, Center for Cancer Research, National Cancer Institute, National Institutes of Health, Bethesda, MD, United States

**Keywords:** haploidentical transplantation, T-cell depleted grafts, T-cell-replete grafts, post-transplantation cyclophosphamide, immune reconstitution, adoptive cell therapy, regulatory T cells, immune evasion

Allogeneic hematopoietic cell transplantation from a related donor who is mismatched for one human leukocyte antigen (HLA) haplotype [HLA-haploidentical transplantation (haploHCT)] has become an effective treatment for patients with high-risk hematologic malignancies, the majority of whom lack an HLA-matched donor. Bi-directional responses to mismatched HLA molecules are associated with high risk of adverse immune reactions driven either by donor alloreactive T cells against recipient tissues [graft-vs.-host disease (GvHD)], or by host alloreactive T cells against the graft (graft rejection). At the same time, donor alloreactive T cells exert a strong graft-vs.-tumor effect because of the mismatch within the unshared HLA haplotype, and this contributes to relapse prevention. The present Research Topic describes how immunological studies pave the way for the development of successful protocols for haploHCT and continuous advancements in the field.

The first safe and effective haploHCT protocol consisted of a myeloablative and immunosuppressive conditioning regimen, the infusion of a “mega-dose” of T-cell-depleted hematopoietic stem cells harvested initially by sedimentation and then through a CD34^+^ positive selection, and no post-transplant immunosuppression. This approach achieved a high rate of engraftment and low incidence of GvHD. The strong graft-vs.-leukemia effect mediated by alloreactive NK cells was unveiled in this setting. However, infection-related mortality was high because donor post-transplant immune reconstitution was slow. Aversa et al. review how T-cell depleted haploHCT has evolved in order to improve outcomes. One approach is to selectively deplete the graft from T-cell subpopulations that can be alloreactive, such as αβ^+^ T cells or CD45RA^+^ naïve T cells. Other groups have developed adoptive cell therapies that aim at boosting immunity against pathogens and malignant cells while controlling adverse alloreactions. Zhang and Tey focus on adoptive T-cell therapies that can provide pathogen- or tumor-specific immunity, or broad T-cell immunity. Among them, switch gene-modified T cells are transduced with suicide genes (e.g., HSV-derived thymidine kinase or inducible caspase 9 genes) and can be conditionally deleted when GvHD occurs. More recently, the importance of the role of CD4^+^Foxp3^+^ regulatory T cells in inducing tolerance after allogeneic HCT has been appreciated. Mancusi et al. report how adoptive immunotherapy with donor conventional T cells under the control of regulatory T cells prevents GvHD, improves immune reconstitution, and preserves the graft-vs.-leukemia effect after haploHCT in the absence of post-transplant immunosuppression.

A strong innovation in the field has been the development of feasible and effective protocols of unmanipulated (T-cell-replete) haploHCT, based on novel strategies that control T-cell alloreactivity and induce T-cell tolerance. One main protocol of T-cell-replete haploHCT is based on the modulation of T-cell immunity using granulocyte colony-stimulating factor-primed grafts, anti-thymocyte globulin, and intensive post-transplant immunosuppression. Chang et al. report further improvements of this protocol, that include the definition of parameters to identify patients with higher risk of post-transplant complications, interventions to overcome poor graft function, and the prophylaxis and treatment of viral infections and relapse.

The second main approach to T-cell-replete haploHCT is the administration of high-dose cyclophosphamide following graft infusion [post-transplantation cyclophosphamide (PTCy)], which allows for the immunomodulation of alloresponses while sparing donor hematopoietic stem cells. Current trials of haploHCT with PTCy are associated with high engraftment rates and low incidences of severe acute and chronic GvHD as well as non-relapse mortality. This approach has become widely adopted over the last several years, but the mechanism by which PTCy prevents GvHD remain incompletely understood. Kato et al. describe the work performed in the 1980s and early 1990s in murine skin allografting models that was instrumental in developing PTCy clinically and established the first model of understanding for how PTCy may work to control alloreactive responses. An adaptation of this model has since become widely accepted in the field. Nunes and Kanakry describe limitations of this model and recent work that challenges this model and provides the framework for a new model of understanding of the mechanisms by which PTCy prevents GvHD. Williams et al. describe the successful and increasingly widespread application of the PTCy approach to HLA-matched related or unrelated donor transplantation platforms, building on the success in haploHCT.

The above protocols of haploHCT variously affect the reconstitution of functional immune cell subsets after transplant, which impacts on outcomes. Zaghi et al. highlight the role of innate lymphocytes, such as NK cells, innate lymphoid cells, and γδ T cells, which tend to reconstitute faster than adaptive B and T cells and could provide early protection against infections and relapse. However, results in patients with unfavorable prognosis, such as those with chemo-resistant disease, are still unsatisfactory, and relapse is still a major cause of treatment failure. Rovatti et al. discuss the mechanisms of immune evasion in allogeneic HCT, such as genomic loss or downregulation of HLA molecules, up-regulation of T-cell inhibitory ligands, release of mediators of T-cell exhaustion, and inhibition of the release of pro-inflammatory cytokines. Among them, the genomic loss of the mismatched HLA haplotype expressed by leukemic cells (known as “HLA loss”) has been detected in ~30% of relapses after haploHCT compared with 5–10% in unrelated donor HCT, possibly reflecting the strong pressure exerted by donor T cells on the mismatched HLA haplotype. Baumeister et al. provide an exhaustive overview of the current protocols for haploHCT, the post-transplant reconstitution of immune cell subpopulations, and the perspectives to further improve outcomes.

The present Research Topic reports how haploHCT has become an effective and widespread treatment so much that survival rates are similar to those after HLA-matched sibling or unrelated donor HCT. Moreover, haploHCT is a feasible platform for further improvements, including the combination with innovative immunotherapies such as bi-specific antibodies and CAR-T cells and CAR-NK cells.

## Author Contributions

AM, CK, and AP wrote the manuscript.

## Conflict of Interest

The authors declare that the research was conducted in the absence of any commercial or financial relationships that could be construed as a potential conflict of interest.

